# Male sperm storage impairs sperm quality in the zebrafish

**DOI:** 10.1038/s41598-021-94976-x

**Published:** 2021-08-17

**Authors:** Silvia Cattelan, Clelia Gasparini

**Affiliations:** Department of Biology, University of Padova, Via U. Bassi 58/B, 35131 Padova, Italy

**Keywords:** Behavioural ecology, Evolution

## Abstract

Variation in sperm traits is widely documented both at inter- and intraspecific level. However, sperm traits vary also between ejaculates of the same male, due for example, to fluctuations in female availability. Variability in the opportunities to mate can indeed have important consequences for sperm traits, as it determines how often sperm are used, and thus the rate at which they are produced and how long they are stored before the mating. While being stored within males’ bodies, sperm are subjected to ageing due to oxidative stress. Sperm storage may significantly impair sperm quality, but evidence linking male sperm storage and variation in sperm traits is still scarce. Here, we tested the effect of the duration of sperm storage on within-male variation in sperm traits in the zebrafish, *Danio rerio*. We found that without mating opportunities, sperm number increased as storage duration increased, indicating that sperm continue to be produced and accumulate over time within males without being discharged in another way. Long sperm storage (12 days) was associated with an overall impairment in sperm quality, namely sperm motility, sperm longevity, and sperm DNA fragmentation, indicating that sperm aged, and their quality declined during storage. Our results confirm that male sperm storage may generate substantial variation in sperm phenotype, a source of variation which is usually neglected but that should be accounted for in experimental protocols aiming to assay sperm traits or maximise fertilization success.

## Introduction

The huge variation observed in sperm traits across species has been widely documented and associated with different evolutionary trajectories determined by differences in ecology and mating systems. Such variation also exists at the intraspecific level and has been reported in many taxa^1–3^. This intraspecific variation has been mainly attributed to differences among populations, male genetic background, developmental environment or mating tactics^4–8^. The latter explains, for example, the large differences in sperm quality and number among males adopting alternative mating tactics such as territorial and sneaker males, where different sperm traits are favoured under different mating circumstances^9,10^. There is also evidence of significant variation across ejaculates of the same males, so that variation exists both in the number of sperm produced and in their quality even when produced from the same male. However, the magnitude and underlying causes of this intra-male variability remain mostly elusive^1,3,11^.

Variation across ejaculates of the same males has been associated with non-genetic variation in sperm phenotype due to variation in male condition, male age, the effect of the social environment, or their interaction^12^. Males in bad condition, due for example to a period of restricted/poor diet, produce less and low-quality sperm as the resources to invest in spermatogenesis are limited (reviewed by^13^). Sperm quality and production also change during the lifespan of the male, due to male ageing, e.g.^14–16^. The social environment is perhaps the most common factor associated with intra-male sperm variation. As one of the strongest selective pressure on sperm form and function, social environment, mainly in the form of sperm competition, has been found to account for within-male variation in sperm traits in several species, e.g.^17–20^. For example, males that perceive a high level of sperm competition, such as males exposed to a male-biased environment, can adaptively adjust the phenotype of their sperm to maximise their reproductive output, e.g.^21,22^ but see^23,24^. An example comes from the wild house mice (*Mus musculus domesticus*) in which males reared under high male density adaptively increase their sperm production^25^.

Another source of variability linked to social environment is female availability, and hence, mating frequency. Fluctuations in mating opportunities is a key factor that can account for profound variation across ejaculates of the same males^26^. Indeed, the frequency at which a male mates will determine how long his sperm will be stored within his body, i.e. the duration of the temporal separation between sperm maturation and sperm release. While being stored in the male, sperm are exposed to oxidative stress, which is due to the accumulation of reactive oxygen species (ROS). Sperm cells have no or little DNA repair machinery and few antioxidant molecules (reviewed by^27^), which make them particularly susceptible to damages mediated by ROS generated during sperm storage^28^. Male sperm storage is thus linked to sperm cellular ageing, called post-meiotic sperm ageing^28,29^.

The duration of sperm storage inside the male affects sperm quality on different levels, and ultimately generates intraspecific variation in sperm phenotype that can have inter-generational epigenetic effects^30^. The stress of prolonged sperm storage by the male has detrimental effects on sperm quality and consequently on fertilization ability^31^, and is well documented in reproductive biology (reviewed in^32–34^). As an example, the WHO guidelines for assisted reproductive techniques in humans recommend no more than 7 days of sexual rest before collecting ejaculates for assays or being used for fertilization^35^. A meta-analysis showed that sexual rest of more than 5 days negatively affects a variety of sperm parameters, including sperm motility, viability and DNA integrity^36^, which in turn impact clinical outcomes of assisted reproductive technologies^33^.

Growing evidence that sperm storage affects sperm phenotype comes mainly from reproductive biology studies, but there is surprisingly limited evidence that demonstrates a link between male sperm storage and intraspecific variation in sperm traits, probably due to the difficulty of obtaining repeated measures in the same male. Notably, exceptions are found in studies in the guppy (*Poecilia reticulata*), in which variation in sperm storage duration has been associated with a decline in both sperm quality^37^ and inter-generational effects^30^.

Here, we explore the magnitude of within-male variation in sperm quality and quantity due to sperm storage, by manipulating the length of time sperm remain in storage within the male in the zebrafish *Danio rerio*. To this end, we sexually deprived males of females and collected and analysed ejaculates from the same male after different periods of time. We first tested whether males keep producing sperm over time, which may indicate that males do not possess a way to avoid sperm to accumulate and age, during storage. Then we tested whether sperm performance (percentage of motile sperm, sperm swimming velocity, trajectory, and longevity) and the amount of sperm DNA fragmentation are impaired during storage, which would indicate that sperm age over time.

## Material and methods

### Fish maintenance

Zebrafish used in this experiment were Tubingen descendants, which were raised and maintained at the Zebrafish facility (University of Padova, Italy). Fish were maintained under standard laboratory conditions (12:12 light–dark cycle; water temperature 28 ± 1 °C) at equal sex-ratio (16 individuals/tank) in 4 L tanks (Muller-Pfleger recirculating system) provided with artificial plants. Fish were fed ad libitum twice per day with dry food and live brine shrimps (*Artemia salina*).

### Experimental overview

Males used for the experiment were of the same age (9 months old) to avoid confounding male age and sperm storage effects^15,28^. We performed a paired experimental design, where sperm were assessed twice from each male that experienced two experimental conditions in randomized order, to account for the variability among males in sperm production and quality^38,39^. Experimental males were initially stripped to empty their sperm reserves before starting the trial. After this initial stripping, each male was individually isolated in a 1.5 L tank and assigned to one of the experimental conditions (i.e. period of sexual rest: 4, 7 or 12 days). The day after the end of the assigned period of sexual rest males were anaesthetised and sperm collected to perform sperm assays (sperm number, motility and viability, see below). Each male was then individually isolated as above and assigned to a second experimental condition (different from the first one), followed by sperm collection and sperm assays as above. We used 36 males in total and each male was tested twice under two different experimental conditions (4 and 7 days: 20 males; 4 and 12 days: 16 males). On a different set of males (N = 28), we also tested the effect of sperm storage on sperm DNA fragmentation, but in this case each male experienced only one of the three possible experimental conditions (i.e. were tested after either 4, 7, or 12 days of sperm storage). For logistical reasons, the experiment was performed in 5 different blocks, with each block consisting of 4–8 males measured in the same day.

### Sperm collection

Sperm collection was performed following an established protocol^38^. Briefly, each male was anaesthetized in a water bath of Tricaine mesylate (MS-222, 0.15 g/L) and placed under a stereomicroscope (ZEISS Stemi 2000-C). The genital area of the male was gently dried to avoid sperm activation by water. Sperm were collected using a 10-μL glass microcapillary (diameter: 0.85 mm) by gently pressing the abdomen of the male. Sperm volume was measured from the microcapillary before releasing the sperm into 50 μL of Hank’s solution maintained in ice^40^. All sperm assays were performed within 15 min from sperm collection.

### Sperm motility

For each assay, 1 μL of diluted ejaculate was activated with 2.5 μL of water on a 12-well slide (MP Biomedicals) coated with a 1% polyvinyl alcohol to prevent sperm from sticking to the glass slide. The slide was immediately covered with a coverslip and sperm motility parameters were measured using a computer-assisted sperm analyser (CEROS, Hamilton-Thorne). We assessed a minimum of 100 sperm cells per ejaculate (except for one ejaculate for which we tracked 65 sperm cells) with an average number of tracked cells of 551.77 ± 29.79 (mean ± SE). We measured sperm velocity (VCL, μm/s), trajectory (measure of path curvature: LIN, linearity), motility (proportion of motile cells over the total) and sperm longevity. Sperm longevity was measured following Poli et al^38^ by recording the time (in seconds) from activation until ≥ 80% of sperm in the field of view were immotile. Sperm velocity parameters were assessed at the time of sperm activation (t0) and 30 s post-activation (t30) as the decline over time in sperm motility occurs largely within 30 s from activation^38,41^. For each ejaculate, we measured sperm motility twice or three times, and calculated the repeatability of sperm motility parameters on the subset measured three times (N = 40).

### Sperm viability and number

Sperm viability and sperm number were estimated using Luna-FL Dual Fluorescence sperm Cell Counter (Logos Biosystems). Following manufacturer instructions, sperm were diluted in Hank’s solution to obtain an optimal sperm concentration for the count. Sperm viability was assessed by dying sperm with a membrane-permeant nucleic acid stain (acridine orange) which labelled live sperm in green, and a membrane-impermeant stain (propidium iodide) which labelled dead or damaged sperm in red. We assessed a minimum of 1000 sperm cells per sample (mean ± SE: 6001.40 ± 434.89). Due to logistical problems we did not obtain sperm viability data from 13 samples, thus our final sample size for sperm viability was N = 57, while for sperm number it was N = 70. Repeatability of sperm viability and sperm number was calculated on a subset of ejaculates (sperm viability: N = 28; sperm number: N = 40).

### Sperm DNA fragmentation

**Figure 1:**
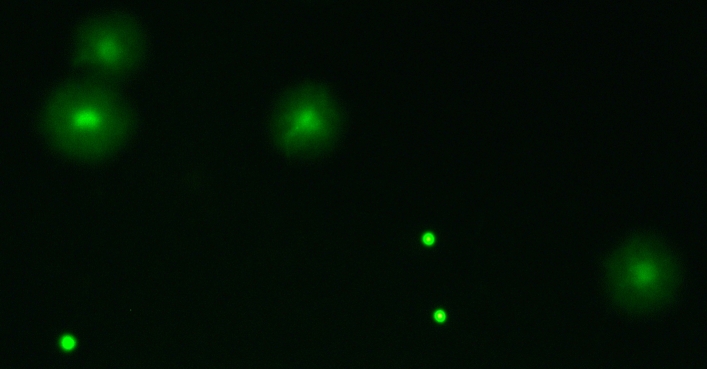
Zebrafish sperm processed with the Halomax-SCD kit. Sperm with fragmented DNA are characterized by the presence of a visible halo, while sperm with intact DNA do not show a halo and retain a compact circular shape.

We assessed sperm DNA fragmentation using the Halomax-SCD kit (Halotech DNA) which is based on the sperm chromatin dispersion (SCD) technique. After sperm were diluted in Hank’s solution at the recommended concentration, we followed the manufacturer’s instructions. To visualize the sperm DNA, the slides were stained with Midori Green Advance (Nippon Genetics) placed under a fluorescent microscope (Leica 5000-B). Sperm with fragmented DNA appeared as large spots with a blurred halo of chromatin dispersion while sperm with intact DNA appeared as small spots with a compact halo of chromatin dispersion (Fig. 1). We counted at least 200 sperm cells for each sample (mean ± SE: 312.86 ± 8.68).

### Statistical analyses

All analyses were performed using R v 4.0.3^42^. Repeatability was calculated on all sperm traits but sperm DNA fragmentation, using the “rptr” package^43^ based on 1000 boostrap replicates. For repeatability, models were fit using a Gaussian distribution for all sperm traits (including sperm motility and viability expressed as percentage), with the exception of sperm longevity for which was used a Poisson distribution. Repeatability of sperm traits was significant for all traits analyzed (see Supplementary Table S1) and we thus used the average value in the subsequent analyses. Linear mixed effect models using “lmer” function in “lme4” package^44^ were used to analyze sperm velocity, linearity, and sperm number. Sperm motility, sperm viability, and sperm DNA fragmentation were analyzed using generalized linear mixed effect models (“glmer” function in “lme4” package) assuming a binomial error distribution and logit link function. Sperm longevity was analyzed using a generalized linear mixed effect model by specifying a Poisson distribution. All the mixed models included the experimental condition (days of sperm storage: 4, 7, 12) as fixed effect and male ID and block (5 blocks in total) as random factors. Each model was checked for normality of residuals by visualizing Q-Q plot of residuals, and for homogeneity of variance by inspecting the residuals vs fitted plot. We calculated *p* values of fixed effects by Type II Wald chi-square tests using the “Anova” function in “car” package^45^. To calculate pairwise effect among the different conditions we performed post-hoc analyses of contrasts with the “lsmeans” in “emmeans” package^46^ using the Tukey method adjusted for multiple comparisons. Means and standard errors are reported.

### Ethical approval

This research was approved by Ethical Committee of the University of Padova (protocol number: 100/2019) and was in compliance with the ASAB guidelines for the Use of Animals in Research and with the ARRIVE guidelines (http://www.nc3rs.org.uk/page.asp?id=1357).

## Results

### Sperm number and motility

**Figure 2:**
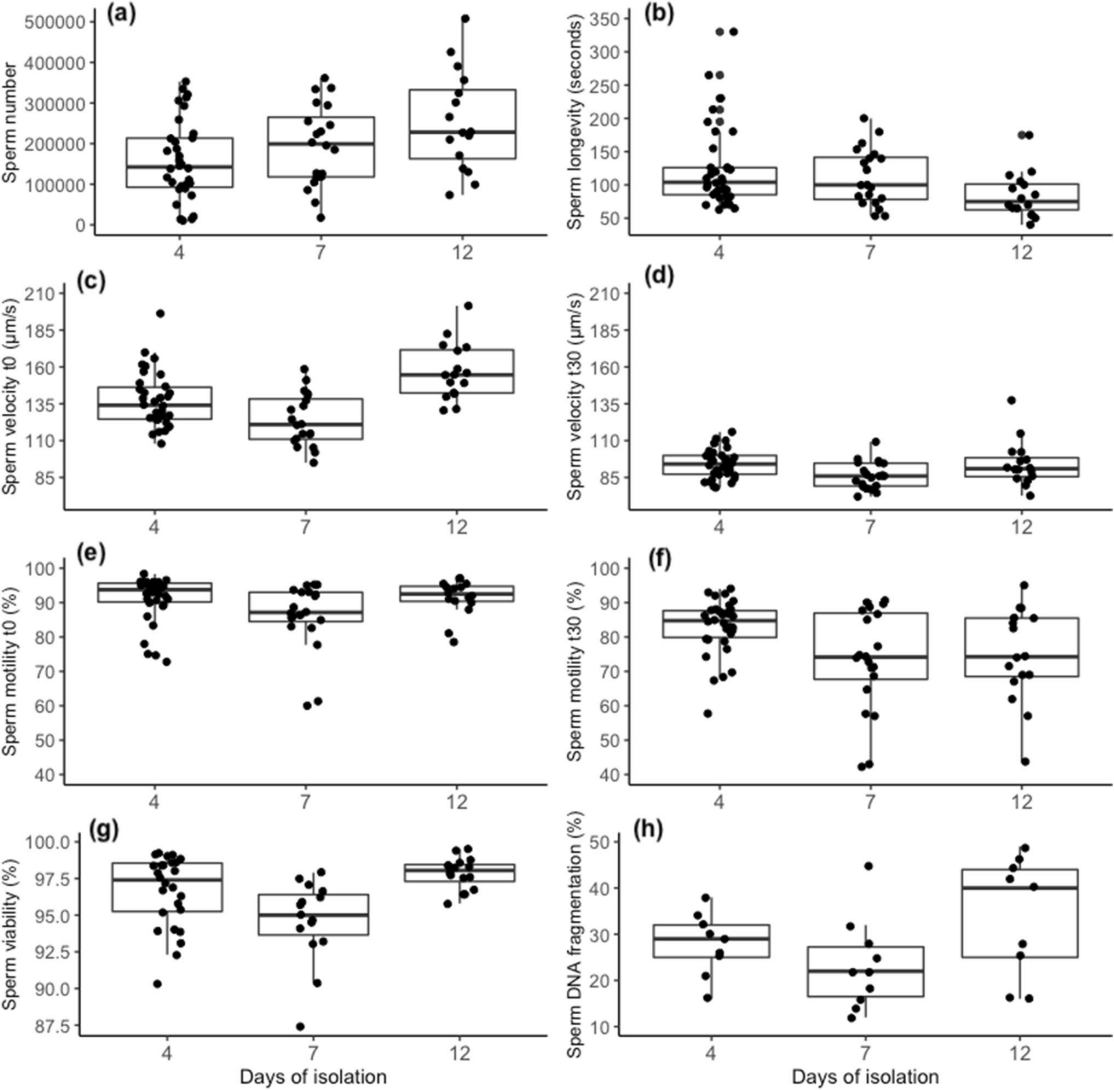
Effects of sperm storage duration on zebrafish sperm traits. Boxplots showing sperm traits in function of sperm storage duration (i.e. day of isolation): 4, 7, 12. Each dot represents a sample [panel (**a**–**f)** 4 days: N = 34, 7 days: N = 20, 12 days: N = 16; panel (**g)** 4 days: N = 26, 7 days: N = 15, 12 days: N = 16; panel (**h**) 4 days: N = 9, 7 days: N = 10, 12 days: N = 9). Note that for sperm DNA fragmentation, each dot represents a single male that has experienced one condition, while for the other sperm traits (panel **a**–**g**) each male experienced two different experimental conditions (4–7 days and 4–12 days). Sperm motility (**e**, **f**), sperm viability (**g**), and sperm DNA fragmentation (**h**) are expressed in percentage for graphical purposes.

Sperm storage significantly affected several sperm traits (Table 1). Overall, sperm storage duration affected sperm number (*p* = 0.001), with sperm number increasing with the increase of duration of sperm storage (Fig. 2a). Sperm storage also affected sperm quality traits, including sperm longevity (*p* < 0.001, Fig. 2b), sperm velocity immediately after sperm activation (t0: *p* < 0.001, Fig. 2c), the proportion of motile sperm (*p* < 0.001, Fig. 2e,f), and sperm linearity (Supplementary Table S2). No effect of sperm storage duration was found for sperm velocity after 30 s from activation (*p* = 0.082, Fig. 2d) or sperm viability (*p* = 0.213, Fig. 2g). Results from the post-hoc tests among the different sperm storage duration (shown in Table 1) revealed that sperm collected from the longest sperm storage (12 days) were significantly faster (Fig. 2c) but lived shorter than sperm from males with 4 or 7 days of sperm storage (Fig. 2b). The proportion of motile sperm was instead highest in sperm from males after 4 days of sperm storage both immediately after activation and after 30 s (Fig. 2e,f).

**Table 1:** Statistical analysis: effects of sperm storage duration on zebrafish sperm traits. Results from linear (a) and general (b) mixed effects models and their associated post-hoc analysis. The analyses were performed on N = 58 samples for sperm viability and on N = 70 samples for all the other variables. *p* values of pairwise differences between the three experimental conditions (sperm storage duration: 4, 7, 12 days) are obtained with the Tukey method adjusted for multiple comparisons. Terms in bold are statistically significant (*p* < 0.05).

Variable	X^2^	*p*	Contrast (days)	Estimate	SE	*p*
Sperm number^a^	13.481	**0.001**	4–7	− 56,250	23,920	0.062
			4–12	− 79,056	28,099	**0.021**
			7–12	− 22,807	36,765	0.810
Sperm longevity^b^	252.46	** < 0.001**	4–7	− 0.05	0.03	0.227
			4–12	0.57	0.04	** < 0.001**
			7–12	0.62	0.05	** < 0.001**
Sperm velocity (t0)^a^	14.892	** < 0.001**	4–7	10.80	5.22	0.112
			4–12	− 16.30	6.03	**0.027**
			7–12	− 27.10	7.84	**0.005**
Sperm velocity (t30)^a^	5.011	0.082	4–7	7.05	3.29	0.096
			4–12	0.48	3.78	0.991
			7–12	− 6.57	4.90	0.386
Sperm motility (t0)^b^	19.157	** < 0.001**	4–7	0.27	0.09	**0.006**
			4–12	0.30	0.10	**0.006**
			7–12	0.03	0.13	0.963
Sperm motility (t30)^b^	148.04	** < 0.001**	4–7	0.54	0.07	** < 0.001**
			4–12	0.59	0.06	** < 0.001**
			7–12	0.05	0.09	0.814
Sperm viability^b^	3.094	0.213	4–7	0.01	0.03	0.980
			4–12	0.08	0.04	0.188
			7–12	0.07	0.05	0.397

### Sperm DNA fragmentation

Sperm storage significantly affected sperm DNA fragmentation (GLMM: *χ*^2^ = 90.310, *p* < 0.001, Fig. 2h), with males after 12 days of sperm storage having more sperm with fragmented DNA than males after 4 and 7 days of sperm storage (post-hoc tests, 4–7 days: *p* = 0.999, 4–12 days: *p* < 0.001, 7–12 days: *p* < 0.001).

## Discussion

Sperm temporarily stored by males before ejaculation are vulnerable to senescence starting as soon as they are produced^28,29^, and can accumulate damages that in turn can impair sperm performance and also their DNA^28^. Here we tested whether the duration of sperm storage within the male affected sperm production and quality in sperm of male zebrafish, by using a repeated measures design to account for intrinsic differences among males in sperm production and quality. While we found a significant increase in the number of sperm produced as the duration of storage increases, the overall quality of sperm decreased. Our results thus suggest that males facing a prolonged period of sperm storage have more sperm available for matings, but those sperm are of lower quality.

We found that sperm number increased with sperm storage, indicating that in this species sperm are continuously produced (at least over the timespan we considered, 12 days) and accumulate in the testes. Thus, if sperm are not released often (e.g. due to low mating opportunities), new sperm keep accumulating in the male along with previously stored sperm.

As expected by theory of sperm ageing^28,29^, we observed that a prolonged period of storage was associated with an overall decrease in sperm quality, indicating that sperm age during storage. We found that sperm motility and sperm longevity were negatively affected by storage. These findings are in agreement with evidence previously reported both in fish and other taxa^47,48^, including humans^36^, indicating that sperm age over time while being stored in males. Extensive evidence of sperm ageing on sperm performance comes from another cyprinodont, the guppy (*Poecilia reticulata*), in which prolonged sperm storage significantly impairs sperm velocity^30,37^. Our results show that males experiencing long sperm storage have sperm with lower motility and lower longevity, but those sperm swim faster immediately after activation (but no longer after 30 s). This initial boost in sperm velocity might, at least partially, provide a fertilization benefit during sperm competition (usually higher sperm velocity is associated with higher competitive fertilization ability in externally fertilizing fish^49,50^), but the relative contribution of velocity, motility, and longevity in competitive fertilization is yet to be determined in this species, and thus also the potential benefit of this initial sperm velocity boost. Interestingly, this initial boost in sperm velocity is associated with decreased motility and longevity. One possible explanation is that sperm ageing may reveal a potential, previously hidden^51^, trade-off between sperm velocity and longevity^52^. Evidence of such a trade-off has been found in many species, e.g.^53–55^. As recently suggested by Reinhardt and Turnell^31^, this trade-off could be mediated by sperm metabolic rate. A higher metabolic rate could provide an instant boost in sperm swimming speed at the expense of sperm lifespan due to increased oxidative stress. In rodents, it has indeed been shown that faster sperm may suffer increased DNA fragmentation^56^ (but see^57^). In line with this, we found that prolonged sperm storage was associated with fast sperm, decreased longevity, and increased level of sperm DNA fragmentation. This suggests that sperm cells may have been directly affected by oxidative stress during sperm storage within the males, which would ultimately lead damages to sperm DNA. This finding is in agreement with previous studies in mammals, including humans^36^, and in particular with results from a previous study in the zebrafish, where males producing faster sperm sired offspring with lower survival^41^, which is probably due to increased DNA damage in sperm^24^. It is worth noting that DNA damage due to oxidative stress likely consists of single-strand DNA breaks, which probably do not prevent sperm to swim and fertilize eggs^58^. The fact that we did not find a significant effect of sperm storage duration on sperm viability reinforces the idea that sperm with damaged DNA are indeed viable and may be capable to fertilize the eggs. In rainbow trout, sperm with DNA damages due to oxidative stress retain fertilization ability, but embryo development and late survival are strongly impacted^59^. Also, the association we found between sperm storage and sperm DNA fragmentation can explain inter-generational effects found in the guppy due to male sperm storage^30^. This suggests that also in the zebrafish, sperm ageing due to male sperm storage may have the potential to generate variance in offspring fitness via inter-generational plasticity, possibly mediated by the DNA fragmentation, but this remains to be tested.

In some animals, strategies have evolved to limit the negative consequences of sperm ageing during male storage and those strategies include getting rid of old sperm, by sperm discharge or sperm reabsorption^28,29^, which has been observed for example in many species of birds^60^ and non-human primates^61^. Similar to other fish species^12,37^, we found that in the zebrafish sexual rest increases sperm number but decreases sperm quality, indicating that males do not possess a way to avoid sperm to accumulate and age, during storage. The zebrafish is a highly social species that lives in shoals^62^ and mates throughout the year, with females spawning every 1–2 days^63^. Although some variation in sex ratio and density may occur between the wet and dry seasons^64^, which may lead males to store their sperm for a few days, males usually mate mutilple times within the same day^62^. On one hand, high mating opportunities may allow males to release sperm frequently, thus minimizing the need for the evolution of a strategy to avoid the consequences of long sperm storage. On the other hand, since under high mating availability and sperm competition (the competition between sperm from different males^65^) males producing more sperm usually fertilize more eggs^66^, males may have been selected for continuously producing sperm to avoid sperm limitation even at the cost of sperm quality^55^. Moreover, it is worth noting that in our study we individually isolated males during sexual rest to avoid the confunding effects of social environment, which could have made difficult the interpretation of results. For example, it has been shown that male-male competition affects sperm phenotype (and in particular morphology and DNA fragmentation^24^) and early embryo quality^41^. However, keeping males isolated prevents us to exclude that the effects of stress associated with social isolation could have exacerbated the effect of sperm storage we unravel in our study. Further experiments are needed to shed light on the role of social environment, such as the presence of rival males and/or females, on patterns of sperm ageing during storage.

In conclusion, we found that sperm ageing mediated by sperm storage within males generates substantial variation in sperm phenotype, a source of variation which is usually neglected but that should be accounted for in experimental designs. This is particularly relevant in the zebrafish as this species serves as a model organism in a large variety of research, where optimizing and standardizing fertilization is crucial. Moreover, when testing for sperm quality, researchers should consider standardizing the duration of sperm storage to obtain more reliable and repeatable results on sperm production and quality, whatever the context is. This could be easily done by manually stripping males to deplete their sperm reserves at a specific time point prior to the ejaculate collection.

## Supplementary Information

Supplementary Information 1.

Supplementary Information 2.

Supplementary Information 3.

## Data Availability

The data and the code associated with this study are available as supplementary materials.
