# Supplementary material for: Male sperm storage impairs sperm quality in the zebrafish
Source: Sci Rep. 2021 Aug 17;11:16689. doi: 10.1038/s41598-021-94976-x (PMC8371167; doi:10.1038/s41598-021-94976-x)
Supplement: Supplementary file 3 — Supplementary Information 3. [file 41598_2021_94976_MOESM3_ESM.pdf]

Male sperm storage impairs sperm quality in the zebrafish

Silvia Cattelan & Clelia Gasparini

Supplementary tables

Table S1: Results from the repeatability analyses of sperm traits.

Variable	R	SE	CI 95%	p
Sperm velocity	0.661	0.072	0.507-0.781	**<0.001**
Sperm linearity	0.725	0.066	0.576-0.823	**<0.001**
Sperm motility	0.462	0.096	0.259-0.629	**<0.001**
Sperm longevity	0.523	0.095	0.294-0.667	**<0.001**
Sperm viability	0.516	0.142	0.179-0.736	**0.002**
Sperm number	0.918	0.030	0.841-0.954	**<0.001**

Table S2: Effect of sperm storage on sperm linearity immediately after activation (t0) and after 30 seconds (t30). P-values of contrasts were obtained with Tukey method adjusted for multiple comparisons. Values in bold are statistically significant ($p<0.05$).

Variable	X^2	p	Contrast	Estimate	SE	p
			4-7 days	4.64	0.90	**<0.001**
Sperm linearity (t0)	35.128	**<0.001**	4-12 days	3.03	1.05	**0.018**
			7-12 days	-1.62	1.38	0.478
			4-7 days	7.01	1.56	**<0.001**
Sperm linearity (t30)	44.710	**<0.001**	4-12 days	9.42	1.77	**<0.001**
			7-12 days	2.41	2.23	0.538
